# *Flavivirus* RNA-Dependent RNA Polymerase Interacts with Genome UTRs and Viral Proteins to Facilitate *Flavivirus* RNA Replication

**DOI:** 10.3390/v11100929

**Published:** 2019-10-10

**Authors:** YanPing Duan, Miao Zeng, Bowen Jiang, Wei Zhang, Mingshu Wang, Renyong Jia, Dekang Zhu, Mafeng Liu, Xinxin Zhao, Qiao Yang, Ying Wu, ShaQiu Zhang, YunYa Liu, Ling Zhang, YanLing Yu, Leichang Pan, Shun Chen, Anchun Cheng

**Affiliations:** 1 Research Center of Avian Disease, College of Veterinary Medicine, Sichuan Agricultural University, Wenjiang District, Chengdu 611130, Chinam18328066380@163.com (W.Z.); mshwang@163.com (M.W.); cqrc_jry@163.com (R.J.); liumafengra@163.com (M.L.); xxinzhao@sicau.edu.cn (X.Z.); yangqiao721521@sina.com (Q.Y.); yingzi_no1@126.com (Y.W.); shaqiu86@hotmail.com (S.Z.); yunnyaaliu@163.com (Y.L.); zl97451@126.com (L.Z.); yanling3525@163.com (Y.Y.);; 2 Institute of Preventive Veterinary Medicine, College of Veterinary Medicine, Sichuan Agricultural University, Wenjiang District, Chengdu 611130, China; zdk24@sicau.edu.cn; 3 Key Laboratory of Animal Disease and Human Health of Sichuan Province, Wenjiang District, Chengdu 611130, China

**Keywords:** *flavivirus*, viral RNA replication, RNA-dependent RNA polymerase

## Abstract

Flaviviruses, most of which are emerging and re-emerging human pathogens and significant public health concerns worldwide, are positive-sense RNA viruses. *Flavivirus* replication occurs on the ER and is regulated by many mechanisms and factors. NS5, which consists of a *C*-terminal RdRp domain and an *N*-terminal methyltransferase domain, plays a pivotal role in genome replication and capping. The C-terminal RdRp domain acts as the polymerase for RNA synthesis and cooperates with diverse viral proteins to facilitate productive RNA proliferation within the replication complex. Here, we provide an overview of the current knowledge of the functions and characteristics of the RdRp, including the subcellular localization of NS5, as well as the network of interactions formed between the RdRp and genome UTRs, NS3, and the methyltransferase domain. We posit that a detailed understanding of RdRp functions may provide a target for antiviral drug discovery and therapeutics.

## 1. Introduction

Flaviviruses, which belong to the *Flavivirus* genus, and *Flaviviridae* family, are globally significant arthropod-borne viruses that cause disease in hundreds of millions of people annually across half of the world; flaviviruses include Dengue virus (DENV, serotypes 1–4), Zika virus (ZIKV), West Nile virus (WNV), Japanese encephalitis virus (JEV), yellow fever virus (YFV), and tick-borne encephalitis virus (TBEV) [1].

Flaviviruses are enveloped viruses with a single-stranded RNA genome, composed of one large open reading frame (ORF), flanked at both its 5′ and 3′ ends by short noncoding sequences termed untranslated regions (UTRs); the *flavivirus* genome also contains a type I cap structure (m^7^GpppAmp) at its 5′ end, and characteristically lacks a poly(A) tail at its 3′ end. The virus enters the host cell by receptor-mediated endocytosis and is internalized, following which the endosome is acidified. The fusion of viral and vesicular membranes allows the release of genomic RNA that serves as the template for translation into the cytoplasm. The ORF is translated at the rough endoplasmic reticulum (ER) membrane and encodes a long single polyprotein that is co- and posttranslationally processed by viral and host cellular proteases, yielding the structural proteins C, prM, and E, and seven nonstructural (NS) proteins (NS1, NS2A, NS2B, NS3, NS4A, NS4B, and NS5). The NS proteins cooperate with an array of host factors to form a membrane-bound replication complex (RC) where viral RNA (vRNA) synthesis takes place. NS5, the largest and most conserved protein among flaviviruses, harbors a C-terminal RNA-dependent RNA polymerase (RdRp) domain and an N-terminal RNA methyltransferase domain that are indispensable for viral RNA synthesis [2]. In addition, the RdRp has been shown to be important for viral replication and RNA synthesis.

Here, we emphasize the process of viral genome biogenesis within the replication complex and discuss molecular interactions between the RdRp and other viral proteins and genomic RNA. Knowledge of these processes may provide new targets for antiviral compound development and the design of vaccine candidates.

## 2. The RdRp Manipulates Viral RNA Replication

Following the translation of viral RNA, *flavivirus* replication occurs in a RC within virus-induced vesicles in the perinuclear region of infected cells; this RC includes viral double-stranded RNA, nonstructural viral proteins, and host cell factors. Although the exact components of the replication complex are not yet well understood, all flaviviral NS proteins have been shown to participate in formation of the replication complex [3,4,5,6,7]. Among members of the RC, NS2A, NS2B, NA4A, and NS4B are transmembrane proteins anchored to the ER membrane [8,9,10]; NS3 is localized to the membrane, where it interacts with NS4B through its C-terminal helicase domain, via the NS3-NS2B complex [9,11]; and NS5 does not have a membrane-associated region but is localized to the membrane via the NS5-NS3 interaction [12]. In addition, recent genetic screens identified multiple host ER-associated enzymatic factors involved in viral replication, e.g., the oligosaccharyltransferase (OST) complex, SEC61A1, SEC63, the signal peptidase complex, and components of the ER-associated protein degradation (ERAD) pathway [13,14]. Although the underlying mechanisms of these host factors remain elusive, these membranous compartments are required for *flavivirus* replication.

**Figure 1:**
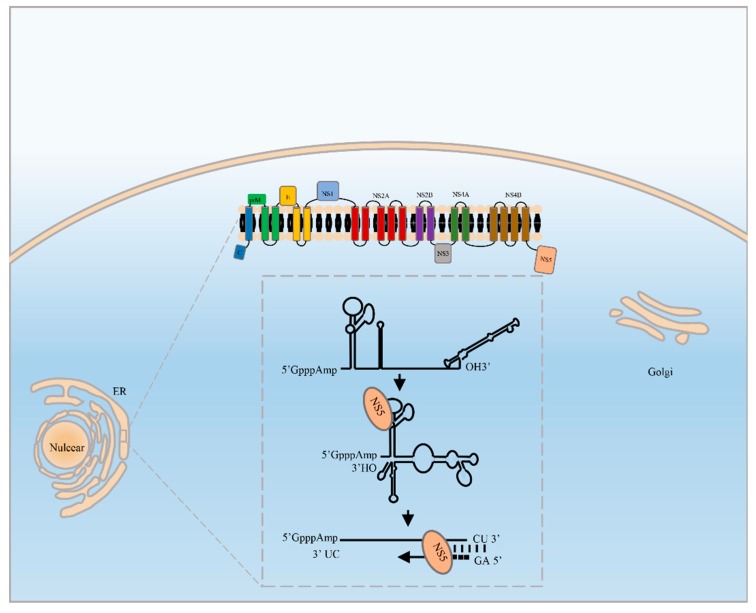
Schema of *flavivirus* genome replication. *Flavivirus* replication occurs on the ER of infected cells in a replication complex (RC), RdRp recognizes the 5′ terminal stem loop A (SLA) via long-range RNA-RNA interactions, bringing the 3′ end close to the 5′ UTR; selectively synthesizes the dinucleotide pppAG over the 3′ terminal RNA template as a short primer; and initiates RNA de novo replication.

Genomic (+) stand RNA is first used as a template from which the RdRp synthesizes a complementary (−) strand RNA, resulting in a double-stranded RNA (dsRNA) replicative intermediate (RI) form. The -RNA then serves as a template for the production of a large excess of positive genomic RNA. The NS3 helicase specifically binds to the conserved 5′UTR sequences 5′-AGUUGUUAGUCU-3′, allowing NS3 to separate the RI into a single strand form in the 3′-5′ direction to release the newly generated viral genome and make the negative strand available as a template for the next round of viral genome synthesis [15]. Several nascent +RNAs are synthesized from –RNA from the RI form in a semiconservative manner, resulting in a 10:1 ratio of positive RNA:negative RNA in the cytoplasm [16]. The RdRp recognizes the 5′-terminal stem loop A (SLA), reaches the site of initiation at the 3′ end via long-range RNA-RNA interactions [17,18,19], and initiates new RNA synthesis from the 3′UTR via a de novo mechanism. The dinucleotide pppAG is selectively synthesized over the 3′ terminal RNA template, which ends in 5′-AG…CU-3′, to form a short primer, and RNA synthesis is then initiated from two nucleotides upstream of the 3′ end of the template [20,21] (Figure 1). The RNA template and incoming and priming nucleotides enter the active site, and the RNA, NTPs, and GTP form a de novo initiation complex. After synthesis of the short primer pppAG, the active center of RdRp switches from a “closed” conformation to an “open” conformation for RNA elongation [22,23]. Newly synthesized viral RNA is packaged, and the immature virion is transported through the host secretory pathway, in which prM is further cleaved to generate a mature virion that is exocytosed from the infected cell.

## 3. The Functional Motifs of the RdRp

### 3.1. Motifs A-E of the RdRp

The architecture of the RdRp is well conserved across flaviviruses, as confirmed by the recently determined crystal structures of the RdRp from WNV, DENV, JEV, and ZIKV. Similar to the architecture of the RdRp domains from other RNA viruses, the *flavivirus* RdRp domain architecture resembles an encircle right hand with three channels (the template entry, dsRNA, and the NTP entry channels), and can be divided into palm, fingers, and thumb subdomains surrounding the active site. A priming loop identified in the thumb subdomain is thought to play a major role in both ensuring correct de novo initiation and providing an initiation platform that stabilizes the de novo initiation complex [2,24,25,26].

**Figure 2:**
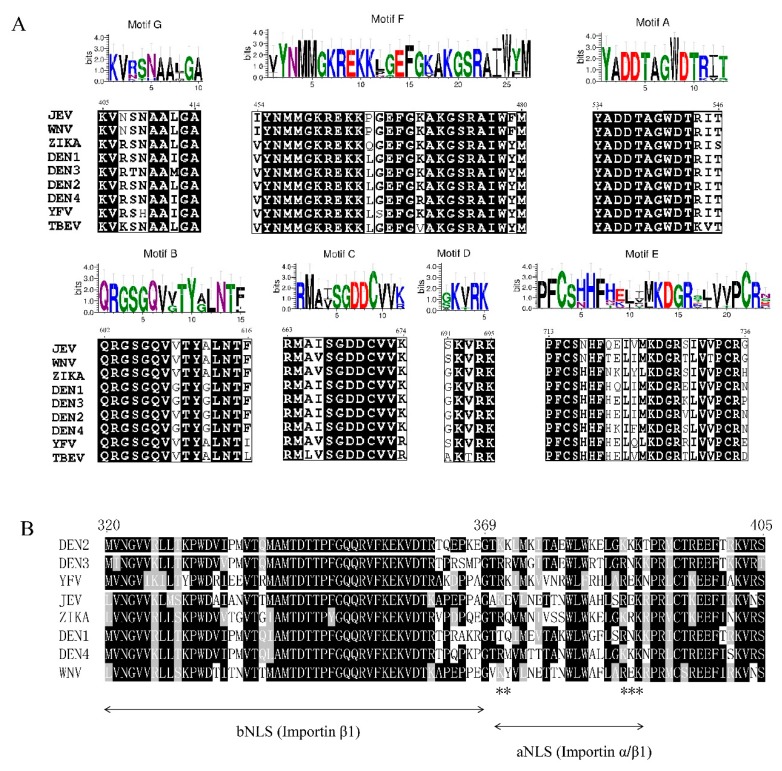
Sequence alignment of *flavivirus* RdRp domain. (**A**) Sequence alignment of RdRp motifs A to G among different flaviviruses. (**B**) Sequence alignment of *flavivirus* bipartite nuclear localization signals. The asterisk shows the key basic residues that serve as functional NLSs.

Seven structural motifs (A to G) responsible for NTP binding and catalysis are shared by all viral RdRps with highly homologous sequences and/or viral RdRps that exhibit structural conservation (Figure 2A). Motifs A and C contain two conserved aspartic acid residues (Asp 533 and Asp 665) involved in the coordination of divalent metal ions for nucleotide polymerization. Motif B helps movement of the template strand in the late stages of transcription [27]. Motif D is related to nucleotide discrimination, and the conserved residue K359 plays an important role in RdRp structural rearrangements required to form the RNA-NTP-UTP complex [28]. Motifs E and C interact with the backbone of the RNA product [29]. Motif F, which consists of the F1, F2, and F3 submotifs, with an F4 submotif observed in the TBEV RdRp [30], is proposed to bind stem loop A prior to viral RNA replication and help stabilize the nascent base pair. The E460D substitution in TBEV motif F was shown to confer resistance to galidesivir, a broad-spectrum RNA virus inhibitor, in cell culture with a 100% survival rate, and no clinical signs were observed in infected mice [31]. Motif G is proposed to regulate access of the ssRNA substrate to the template channel and/or RdRp translocation [32]. In addition, two conserved cavities in the thumb subdomain, cavity A and cavity B, are found in the RdRp domain of DENV. Functional mutagenesis of these two cavities showed that cavity B, but not cavity A, is essential for RNA synthesis. The alanine mutation of L328, W859, and I863 in cavity B decreased the initiation of RNA synthesis potentially by affecting formation of the RNA template-RdRp-NTP complex. Furthermore, the K330A mutation abolished viral replication by reducing the NS3/NS5 interaction [33].

The RNA template entry tunnel and “N” pocket located at the junction of the thumb and palm subdomains are two conserved inhibitor-binding sites in the RdRp domains of both DENV and ZIKV, and a series of inhibitors have been identified [34,35]. NITD107 is targeted to the RNA tunnel and may prevent viral RNA synthesis via competition with the RNA substrate after binding [25]. Based on fragment screening, JF-31-MG46, compounds 27 and 29, and compound 8 of 2,1-benzothiazine 2,2-dioxides were identified to bind in the “N pocket”. These compounds were proposed to hinder RdRp conformational changes during its transition from initiation to elongation, and thus inhibit RdRp activity [36,37,38].

### 3.2. Nuclear Localization Signal of the RdRp

Interestingly, *flavivirus* replication occurs within the cytoplasm of infected cells; however, NS5 can translocate from the cytoplasm to the nucleus. In DENV-2, DENV-3, ZIKV, and YFV, a significant amount of NS5 accumulates within the nucleus, while NS5 in DENV-1, DENV-4, JEV, and WNV is predominantly localized within the cytoplasm of infected cells [39,40,41,42,43]. Nevertheless, WNV_KUN_ NS5 has been demonstrated to localize within the nucleus only in the presence of a specific nuclear export inhibitor [42]. Bipartite nuclear localization signals (NLSs) distributed between the fingers and thumb subdomains comprise an aNLS (residues 369–405), which is recognized by the conventional NLS-binding importin α/β heterodimeric nuclear import receptor, and a bNLS (residues 320–368), which is recognized by importin β1, can target β-galactosidase to the nucleus (Figure 2B), and acts as the binding site for the flaviviral helicase [44,45]. However, in ZIKV, the NS5 NLS is located in the aNLS region and interacts with only importin α [46].

Site mutagenesis of NLS showed that the aNLS, rather than the bNLS, is essential for NS5 nuclear accumulation and viral replication, particularly as the mutation of ^387^KKK^389^ in DENV-2 NS5 abolished NS5 nuclear import and viral production, which suggested the integral role of nuclear NS5 during *flavivirus* infection [47]. In ZIKV, the ^390^KRPR^393^ in the monopartite NLS is necessary to direct NS5 to the nucleus, and its mutation to ^390^ARPA ^393^ changed NS5 localization from the nucleus to the cytoplasm [46]. In addition, the C-terminal 18 amino acids of NS5 regulate the translocation of NS5 between the cytoplasm and nucleus. The P884T mutation resulted in the mislocalization of NS5 to the cytoplasm without compromising viral fitness, and the R888K mutation led to a severely attenuated phenotype, even though NS5 was located in the nucleus [48]. Although a large proportion of NS5 accumulates in the nuclei of infected cells, the role of NS5 in the nucleus has not yet been elucidated. Recent studies have suggested that nuclear NS5 modulates host cell immune responses and virus production [46,47,49] and affects the splicing of antiviral response mRNAs [50]. In infected cells, NS5 is able to bind and degrade the IFN-regulated human transcriptional activator STAT2 to suppress type I interferon signaling in ZIKV [51]. STAT1 is found to colocalized with ZIKV NS5, and overexpressed NS5 is able to upregulate STAT1 related genes, however, this regulation is dampened in response to expression of mislocated NS5 mutant [46].While in DENV, only proteolytically processed NS5 can efficiently mediate STAT2 degradation, particularly when the cellular protein UBR4 binds to NS5, while the unprocessed and processed forms of NS5 can bind NS5 [52,53]. Furthermore, nuclear NS5 is also thought to dampen the IL-8 induction response, resulting in increased virus production [49]. Proteomic and transcriptome analyses of DENV-infected cells showed that nuclear NS5 interacts with the spliceosome U5 snRNP proteins and hijacks the splicing machinery, thus forming an environment less restrictive for viral replication [50]. Analysis of the crystal structure of DENV NS5 showed that these two NLSs are present in the RdRp domain, raising the possibility that the NLS regulates RdRp activity via the rearrangement of NS5 [54].

Conventionally, NS5 translocation is thought to occur through the activities of intracellular nuclear transport proteins and members of the host importin (IMP) superfamily [55]. Knowledge of the interaction between NS5 and IMP has enabled the identification of *flavivirus* NS5 nuclear import inhibitors with novel screening approaches, and many specific small-molecule inhibitors, some of which have apparent antiviral activities, have been analyzed. Ivermectin has been shown to inhibit importin α/β1, leading to a significant decrease in DENV production [56]. *N*-(4-Hydroxyphenyl) retinamide (4-HRP), which can block the recognition of DENV NS5 by the host nuclear import proteins IMP-α/β1 and thus reduces viral RNA levels and titers, is equally effective in protecting against DENV-1-4- and ADE-mediated infection [57,58]. GW5074 targets the host nuclear transport protein IMPα and prevents IMPα heterodimerization with IMPβ1, thereby blocking the ability of IMPα to transport NS5 to the nucleus. Meanwhile, GW5074 at low concentrations has shown strong antiviral activity against DENV-2, ZIKV, and WNV [59].

## 4. The Interactions Between the RdRp and Functional Viral Components

### 4.1. RdRp Binds to the 5′UTR and 3′UTR to Initiate RNA Synthesis

The 5′- and 3′-terminal regions of the *flavivirus* genome consist of multiple RNA sequence elements and tertiary structures that function as cis-acting elements and regulate replication and translation of the viral genome. In particular, the 5′ UTR comprises two stem loops called stem loop A (SLA) and stem loop B (SLB), and SLA has been shown to function as a promoter for RNA synthesis during the replication process [60,61]. The 3′UTR is divided into three regions according to sequence variability (domains I, II, and III), and the 3′SL of domain III is a major determinant of viral RNA replication competence [62]. In addition, complementary sequences present at both ends of the genome are essential for flaviviral RNA synthesis, and at least three pairs of RNA cyclization sequences mediate long-range RNA-RNA interactions. These sequences include the 5′-3′ cyclization sequences (5′-3′CSs), 5′-3′ sequence upstream of the AUG region (5′-3′UAR), and a 5′-3′ sequence downstream of the AUG region (5′-3′ DAR) [63,64,65].

EMSA and footprinting assays documented the interaction between NS5 and the first hairpin element in the 5′ UTR, designated stem loop A (SLA). In this model, specific structures of the SLA, a top loop and a side stem loop, were found to be necessary for genome replication in infected cells and polymerase activity in vitro. Mutations of nucleotides at the top of SLA and in the side stem loop of the SLA element impaired promoter-dependent RNA synthesis, and revertant viruses restored viral replication when specific mutations were introduced at the top loop of SLA [60]. Deletion of SLA within the 5′UTR (DENV) eliminated protein binding. However, deletion of SLB, the 5′CS, or both regions did not affect RNA production [61]. The NS5-SLA interaction is affected by MgCl_2_ and NaCl concentrations in solution, and NS5 is able to bind SLAs from different DENV serotypes, which indicated that NS5 recognizes the overall shape of SLA, as well as specific nucleotides, to form an SLA-polymerase complex [66]. Recently, a 5′-UAR-flanking stem (UFS) element within SLB was demonstrated to play an important role in efficiently recruiting the polymerase to the 5′ end of the *flavivirus* RNA, and a switch-like structure formed by genome cyclization has been shown to regulate dynamic RdRp binding for RNA synthesis [67]. Recently, it was proposed that RNA binding showed no preference between NS5 and RdRp, since both of these proteins bound RNA through the same interacting sequence. Furthermore, the MTase exhibited negligible RNA specificity [68]. Studies on the interaction between the DENV RdRp with the viral genome suggested that the RdRp thumb domain recognizes an ACAG motif. Intriguingly, both the 5′SLA and 3′SL contain the ACAG motif. Site-directed R770A, R771A, R8561, and K841A mutations of RdRp abolished its interactions with both the 3′SL and 5′SLA, suggesting that there is likely a specific RNA-binding site in RdRp. Therefore, a proposed Arg-rich site in the thumb domain of the RdRp could be the RdRp-SLA interaction site [66,68].

Generally, the 3′UTR is structurally subdivided into three autonomously folded regions (domains I–III) that show sequence and structural conservation across members of the genus to various extents; furthermore, the extreme 3′ region is a small hairpin 3′ stem loop (sHP-3′SL) [69,70,71]. Domain I is located downstream of the translation stop codon. In most flaviviruses, domain I appears as a hypervariable sequence followed by two conserved stem loop domains (SL-I and SL-II). While domain II is moderately conserved, domain II in WNV contains only one characteristic dumbbell (DB) structure, while that in DENV-2 contains duplicate DB structures (DBI and DBII). Domain III is defined by two highly conserved terminal genomic functional elements: a short hairpin (sHP) and 3′SL. The top loop of the 3′SL (3′SL-TL) contains a conserved pentanucleotide sequence, 5′-CACAG-3′, with occasional substitutions at the 4th nucleotide. Mutagenesis showed that base pairing and the sequence at the 1st, 2nd, 3rd, and 5th positions, but not the 4th position, of the pentanucleotide are critical for RNA synthesis [72]. The ACAG motif in the 3′SL-TL serves as a cis-element for (-) strand viral RNA synthesis in cells. Deletion of the 3′SL-TL dramatically impaired RdRp complex formation and completely abolished viral RNA replication in BHK-21 cells.

Critical residues in the thumb domain that participate in 3′SL-RdRp interactions were defined by RNA-protein interaction assays. R773, R770, Y838, and K841 likely create a TL RNA-accommodating platform, while Y838 seems to play a key role in side chain stacking during RNA recognition [68]. A previous study showed that RdRp has a narrow template channel that accommodates the 3′ end of an RNA in only a single-stranded form. Thus, the tertiary structure of the 3′ UTR would impair polymerase activity and repress RNA synthesis; thus, structural changes around the 3′ terminal nucleotides seem to be a prerequisite for polymerase initiation [60]. However, the recently attained structure of HCV RdRp in complex with a double-stranded RNA model revealed that removal of a β-hairpin loop that impedes access of the template RNA to the thumb domain in NS5B increased de novo RNA synthesis by >100-fold, promoted RNA binding, and induced significant conformational changes producing an open form, allowing primer-template RNA duplex access to the catalytic core [73]. Taken together, these findings indicate that cooperation between the 3′ SL and RdRp is required for RNA synthesis. 5′-3′-Hybridization not only brings the 3′ end of the RNA near the 5′ promoter, but also plays a role in making the 3′-terminal nucleotides of the viral genome available for the viral RdRp during the initiation of RNA synthesis. Furthermore, the interaction between 3′SL and RdRp facilitates rearrangement of the thumb domain, which provides the template access the active site to initiate de novo RNA synthesis.

An in vitro RNA synthesis assay showed that recombinant full-length NS5 and its truncated RdRp domain alone are capable of initiating both de novo and primer-dependent (3′-end elongation) replication using various RNA templates in vitro [74,75,76,77]. The 3′ 83 nucleotides of JEV were demonstrated to be the minimal RNA template required for RNA synthesis initiation [21]. In vitro DENV RNA synthesis experiments showed that RdRp synthesis requires exogenous viral RNA template containing the 5′- and 3′ UTRs; these UTRs contain conserved elements including the highly conserved 5′-CYC motif, which is complementary to the 3′-CYC motif within the 3′UTR and the 3′-stem loop region [19]. However, although the template can contain plus- or minus-strand 3′-untranslated regions of the genome, a greater amount of +RNA synthesis is observed with the latter RNA template [21].

### 4.2. RdRp Cooperates with NS3 to Facilitate RNA Synthesis

NS3 and NS5 are the two major enzymatic components required for viral RNA unwinding and subsequent RNA synthesis, respectively. How these two enzymes cooperate during viral replication is not known. NS3 contains an N-terminal serine protease domain and a C-terminal ATP/helicase domain [78]. The NS3 helicase domain is further divided into three subdomains. Subdomains I and 2 are associated with ATP binding and/or hydrolysis and interdomain communication and RNA binding, respectively. Subdomain 3 has been suggested to interact with the RdRp [79,80].

The interaction between the flaviviral NS3 and NS5 proteins has been demonstrated by immunoprecipitation assays with cultured cells and yeast two-hybrid studies, and the following interacting regions have been mapped: the C-terminal region of NS3 (residues 303–618) and the N-terminal region of RdRp domain (residues 320–368) [44]. A previous study showed that NS5 stimulates NS3 NTPase and RTPase activities. In an NTPase activity assay, NTPase activity was stimulated by NS5 in a dose-dependent manner. Meanwhile, the RTPase activity of NS3 was shown to be increased in the presence of NS5 [81]. A recent study in ZIKV NS5 showed that its helicase activity is specifically facilitated by the opening of dsRNA with a 3′ overhang through an increase in the dsRNA unwinding velocity, and the mutation of two conserved residues in NS3 that might disrupt the NS3-NS5 interaction, N569A and E573A, leads to significant deficiencies in viral replication [82]. Residues N570 in NS3 and K330 in NS5 are key residues involved in the interaction between NS3 and NS5. The mutation of K330 in NS5 disrupted the NS3-NS5 interaction and completely abolished viral replication, while the N570A NS3 mutation induced the synthesis of low but unsustainable amounts of viral RNA with transfected RNA used as a template [33,80].

Although the NS3-RdRp interaction has been demonstrated in several flaviviruses, the exact function of cooperation between these two enzymatic proteins is unknown. NS5 or the RdRp may facilitate the localization of NS3 close to the entrance of the RNA template tunnel, so that these two catalytic components are in close spatial proximity to enable efficient RNA synthesis.

### 4.3. The MTase Interacts with the RdRp to Regulate RNA Synthesis

NS5, the largest and most conserved *flavivirus* protein, consists of both an MTase involved in capping and the central replication enzyme, the RdRp, naturally fused through a 10-residue flexible linker. A longstanding question, however, has been whether these two domains cooperate to regulate viral replication and cap formation. Intramolecular interactions between the MTase and RdRp were first identified through analysis of the full-length NS5 structure by in silico docking of the WNV MTase domain onto the RdRp domain [2]. The MTase has been proposed to interact with the finger subdomain of the RdRp, primarily through a hydrophobic network that involves P113, L115, and W121 from the MTase and Y350, F466, and P584 from the RdRp. Notably, none of these six residues participate in catalysis, but five out of the six residues are highly conserved across flaviviruses, with the exception of L115, which is moderately conserved [32,83,84].

According to overall crystal structure analysis of *flavivirus* NS5, the MTase domain is stabilized by motif F in the RdRp domain, primarily by the conserved residue F466, which stacks against a pocket on the surface of the MTase domain formed by L115, L126, and P113. The interaction between F446 and P113 is the key feature of the MTase-RdRp interface among *flavivirus* NS5 [29]. Among YFV, ZIKV, and JEV NS5, the residues present at the interface of the MTase and RdRp domains are highly conserved and adopt similar conformations. By comparison, in DENV, the MTase-RdRp interaction utilizes a different interface due to disorder in motif F in the NS5 structure [29,84]. Mediated by the linker, the MTase domain is attached to the backside of the RdRp domain through hydrophobic interactions, which shields the top-right rim of the NTP entry channel. The close proximity of the MTase and RdRp suggests that the orientation of the MTase regulates the newly synthesized viral RNA exiting from the template-binding channel of the RdRp domain [32].

It was recently proposed that two molecules of NS5 inside the asymmetric unit form a dimer in which the MTase domains are oriented away from each other. In the NS5 monomer, the dsRNA exit site of the RdRp and the active site of the MTase face opposite directions in both the DENV and JEV NS5 structures. In contrast, in the NS5 dimer, the MTase and RdRp domains face the same direction [85]. Six independent ZIKV NS5 molecules in the crystallographic asymmetric unit were found to be organized into three quasi-equivalent dimers. Each monomer contributes to the dimer interface, which is composed of two types of intermolecular contacts between MTase-MTase and MTase-RdRp. MTase-RdRp contacts at the dimer interface include residues E155, V156, A159 from the MTase of the first monomer and the following residues from two subdomains of the neighboring NS5 RdRp domain: i) L321, I322, V325 (β11) and G324 and V325 (α10) within the fingers subdomain and ii) G747, I750 (β22-α28 loop) and M871 and R874 and I875 (α33) at the back of the thumb subdomain. In this arrangement, the active site cavities of the MTase domains of two interacting molecules are directly connected, and the two RdRp exit channels remain accessible to the solvent [86].

The impact of the MTase domain on RdRp activity was tested by comparing the RdRp activities of recombinant NS5 and RdRp proteins. Deletion of the ZIKV NS5 MTase domain decreased de novo-initiated RNA products to approximately half the level observed with full-length NS5, especially the products of RNA elongation [83]. Full-length DENV2 NS5 exhibited superior de novo initiation and elongation activities compared to the RdRp domain alone [87]. Substitution of the DENV2 NS5 RdRp with the corresponding region from DENV4 severely attenuated replication in infected cells, as did reverse mutations at K761 and D51 in the DENV2 MTase domain [88]. When mutations to polar or charged residues were introduced into these sites, viral replication levels were significantly affected [89]. The disruption of dimer formation and the MTase-RdRp interface by the R681A mutation of the RdRp drastically affected in vitro RdRp elongation activity [90]. These results indicated that the interaction between the MTase and RdRp is important for polymerase activity, since full-length NS5 has higher polymerase activity than the RdRp alone for efficient viral replication.

MTase-RdRp interactions potentially promote the adoption of different NS5 arrangements to facilitate positioning of the MTase near the site of the recently synthesized dsRNA exit and enable 5′-RNA cap transfer. However, adopting this conformation might be limited in the replication complex due to the multiple protein-protein interactions on the membrane. In the dimeric NS5 structure, the distance from the dsRNA exit of the RdRp of one monomer to the MTase active site of the other monomer is closer than the distance of the RdRp of one monomer to its own MTase; thus, the dsRNA product could more easily access the MTase active site of the neighboring RdRp.

## 5. Conclusions

There are still gaps in our understanding of how the RdRp interacts with viral proteins and genomic RNA to efficiently coordinate their respective functions during RNA synthesis and capping. To date, there are no antiviral therapies available, and limited vaccines for flaviviruses. As the most conserved protein among flaviviruses, the RdRp plays a vital role in viral replication, which allows researchers to utilize this promising target for the development of antiviral inhibitors and therapeutics. In this review, we have attempted to comprehensively cover viral genome biogenesis mediated by the RdRp protein. The RdRp motifs, which are conserved among flaviviruses, participate in catalyzing de novo initiation, NTP binding and new RNA synthesis. In some sections, we emphasized the subcellular localization of NS5 and small-molecule inhibitors developed to target this region. Interestingly, the subcellular localization of NS5 from different flaviviruses varies. To some extent, the NLS within the RdRp domain leads to different subcellular NS5 distributions; however, this sequence is not well conserved, and further understanding of the mechanism of this difference and the distinct functions of nuclear NS5 is required. In addition, during the viral life cycle, RdRp regulates RNA replication via protein-RNA and protein-protein interactions to facilitate the efficient replication of genomic RNA. For example, the RdRp recognizes the initiation site of the genome via an RdRp-UTR interaction, the interaction between RdRp and NS3 promotes NTPase and helicase activity, and the interaction between the RdRp and the MTase is involved in new RNA synthesis. The RdRp is indispensable for *flavivirus* replication because of not only its own polymerase activity, but also its interactions with other viral proteins and RNAs, which leads to efficient genomic RNA replication. However, understanding how these components cooperate and determining whether they interact in a distinct order or ratio requires further study.
